# Imaging alloreactive T cells provides early warning of organ transplant rejection

**DOI:** 10.1172/jci.insight.145360

**Published:** 2021-07-08

**Authors:** Toshihito Hirai, Aaron T. Mayer, Tomomi W. Nobashi, Po-Yu Lin, Zunyu Xiao, Tomokatsu Udagawa, Kinya Seo, Federico Simonetta, Jeanette Baker, Alan G. Cheng, Robert S. Negrin, Sanjiv S. Gambhir

**Affiliations:** 1 Division of Blood and Marrow Transplantation, Stanford University, Stanford, California, USA.; 2 Department of Urology, Tokyo Women’s Medical University, Tokyo, Japan.; 3 Department of Bioengineering,; 4 Department of Radiology,; 5 Molecular Imaging Program at Stanford, and; 6 BioX Program at Stanford, Stanford University, Stanford, California, USA.; 7 Molecular Imaging Research Center of Harbin Medical University, Harbin, China.; 8 Department of Otolaryngology-Head and Neck Surgery,; 9 Division of Cardiovascular Medicine,; 10 Department of Materials Science and Engineering, and; 11 Canary Center at Stanford, Stanford University, Stanford, California, USA.

**Keywords:** Immunology, Transplantation, Organ transplantation, T cells

## Abstract

Diagnosis of organ transplant rejection relies upon biopsy approaches to confirm alloreactive T cell infiltration in the graft. Immune molecular monitoring is under investigation to screen for rejection, though these techniques have suffered from low specificity and lack of spatial information. ImmunoPET utilizing antibodies conjugated to radioisotopes has the potential to improve early and accurate detection of graft rejection. ImmunoPET is capable of noninvasively visualizing the dynamic distribution of cells expressing specific immune markers in the entire body over time. In this work, we identify and characterize OX40 as a surrogate biomarker for alloreactive T cells in organ transplant rejection and monitor its expression by utilizing immunoPET. In a dual murine heart transplant model that has both syngeneic and allogeneic hearts engrafted in bilateral ear pinna on the recipients, OX40 immunoPET clearly depicted alloreactive T cells in the allograft and draining lymph node that were not observed in their respective isograft counterparts. OX40 immunoPET signals also reflected the subject’s immunosuppression level with tacrolimus in this study. OX40 immunoPET is a promising approach that may bridge molecular monitoring and morphological assessment for improved transplant rejection diagnosis.

## Graphical abstract

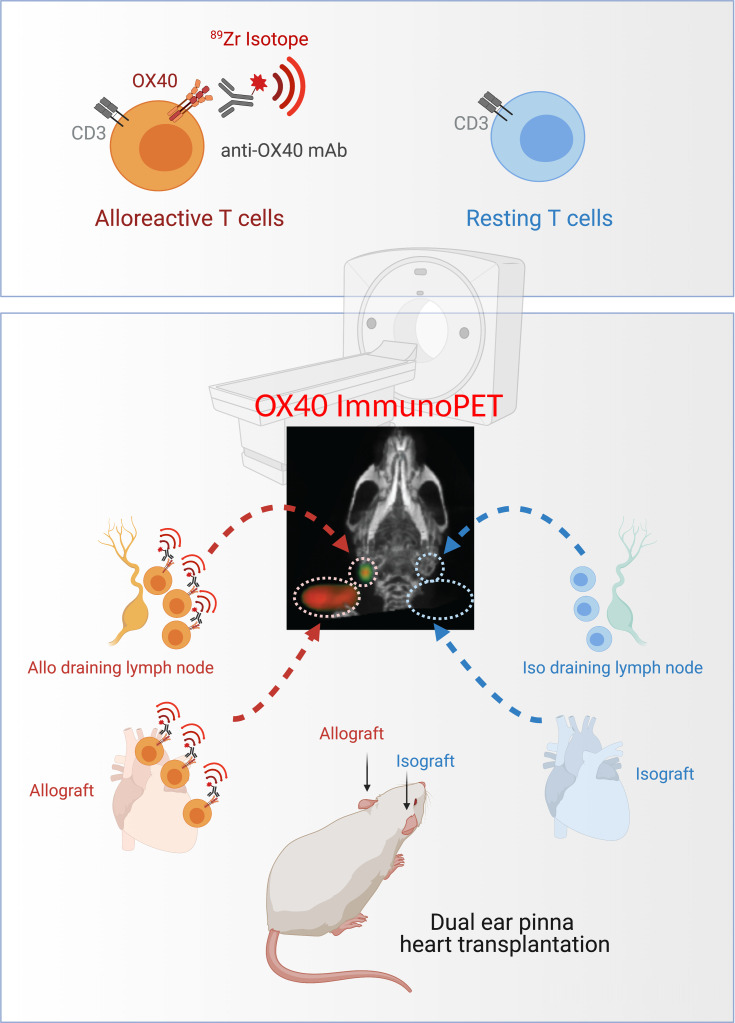

## Introduction

Organ transplantation is a life-saving procedure for the more than 36,500 patients who receive transplants each year in the United States. Despite the introduction of effective immunosuppressant drugs, 40% of heart transplant recipients and 10%–12% of kidney transplant recipients will ultimately experience immunological graft rejection that endangers graft survival (1, 2). Because the risk of organ transplant rejection can be significantly decreased through early detection, patients undergo regular monitoring of graft function and periodic tests that are utilized to detect signs of graft rejection. A variety of biomarkers have been studied for the detection and monitoring of organ transplant rejection, including proteomic, transcriptomic (3–6), and cell-free DNA approaches (7). Transcriptome assessments including multi-gene panels assayed on peripheral blood mononuclear cells (PBMCs) found in patients’ blood samples have shown promise for the identification of severe rejection episodes, though the blood signature might be influenced by other systemic inflammation events. Indeed, AlloMap, which was the first FDA approved test based on this approach, suffers from a low positive predictive value (8). Therefore, patients who show positive results in these sensitive assays also need to undergo tissue biopsies. Conventional biopsies remain the gold standard for diagnosing graft rejection, but they, too, have limitations. These include the potential for transplant damage and complications stemming from the invasive nature of the test. Altogether, there exists a critical need for alternative, minimally invasive tests that can detect organ transplant rejection early and accurately.

Molecular imaging enables whole body, noninvasive, longitudinal monitoring that has the potential to overcome some of the current challenges in monitoring organ transplant rejection. Anatomical imaging utilizing CT, MRI, and ultrasound plays a major role in routine clinical practice for the evaluation of graft viability by measuring size, vascularity, and functionality (9, 10). However, abnormalities identified from these modalities tend to be detected as a result of rejection and fail to capture initial events. Compared with such imaging modalities, molecular imaging has the potential to visualize upstream metabolic and cellular activity that occurs prior to morphological changes. To date, molecular imaging studies have been limited in the organ transplant setting due to the lack of specific biomarkers and molecular probes for monitoring immune cells. The most commonly used molecular imaging probe, fluorine-18-fluorodeoxyglucose ([^18^F]FDG), which provides a measure of local inflammatory activity through uptake of glucose by immune cells (11), was tested in several animal rejection models (12–15). However, in the clinic, lack of specificity makes the diagnosis of organ transplant rejection difficult with this approach (16).

The development of new molecular imaging probes specially designed to target immune cells has the potential to be both highly sensitive and specific for detecting transplant rejection. Molecular imaging of innate immune cells has been reported to enable detection of cardiac allograft rejection through measuring phagocytic activity in macrophages (16) or visualizing myeloperoxidase activity in graft-infiltrating granulocytes (17). ImmunoPET imaging with a radiolabeled antibody against sialoadhesin also demonstrated macrophage infiltration into cardiac allografts (18, 19). Though these approaches could be effective for the detection of macrophages and granulocytes that are frequently observed in damaged tissues, including rejected allografts, it is difficult to distinguish the cause of cell infiltration between immunological rejection and unspecific inflammation such as infection. This discrimination is critical because treatment strategies for rejection and infection greatly differ.

To directly image T cells in vivo, which are central mediators of graft rejection, immunoPET probes targeting major T cell markers such as CD8 (20) or CD3 (21) were developed in previous studies. However, these approaches detect the majority of all T cells in the body irrespective of activation status. We have recently identified OX40 as an imaging biomarker of activated T cells and developed an immunoPET tracer targeting the OX40 receptor. OX40 expression is highly restricted to antigen-specific activated T cells (22, 23), in contrast to other cell surface activation markers (e.g., CD25, CD44, CD69) and secreted markers (e.g., granzyme B, IFN-γ; refs. 24, 25) that are associated with numerous cell types. We have previously established OX40 as an important imaging target in cancer models (26). In this study, we sought to characterize OX40 as a specific biomarker of alloreactive T cells and establish OX40 PET imaging as a method for early detection and monitoring of organ transplant rejection.

## Results

### Establishment and characterization of dual heart transplant rejection model.

**Figure 1:**
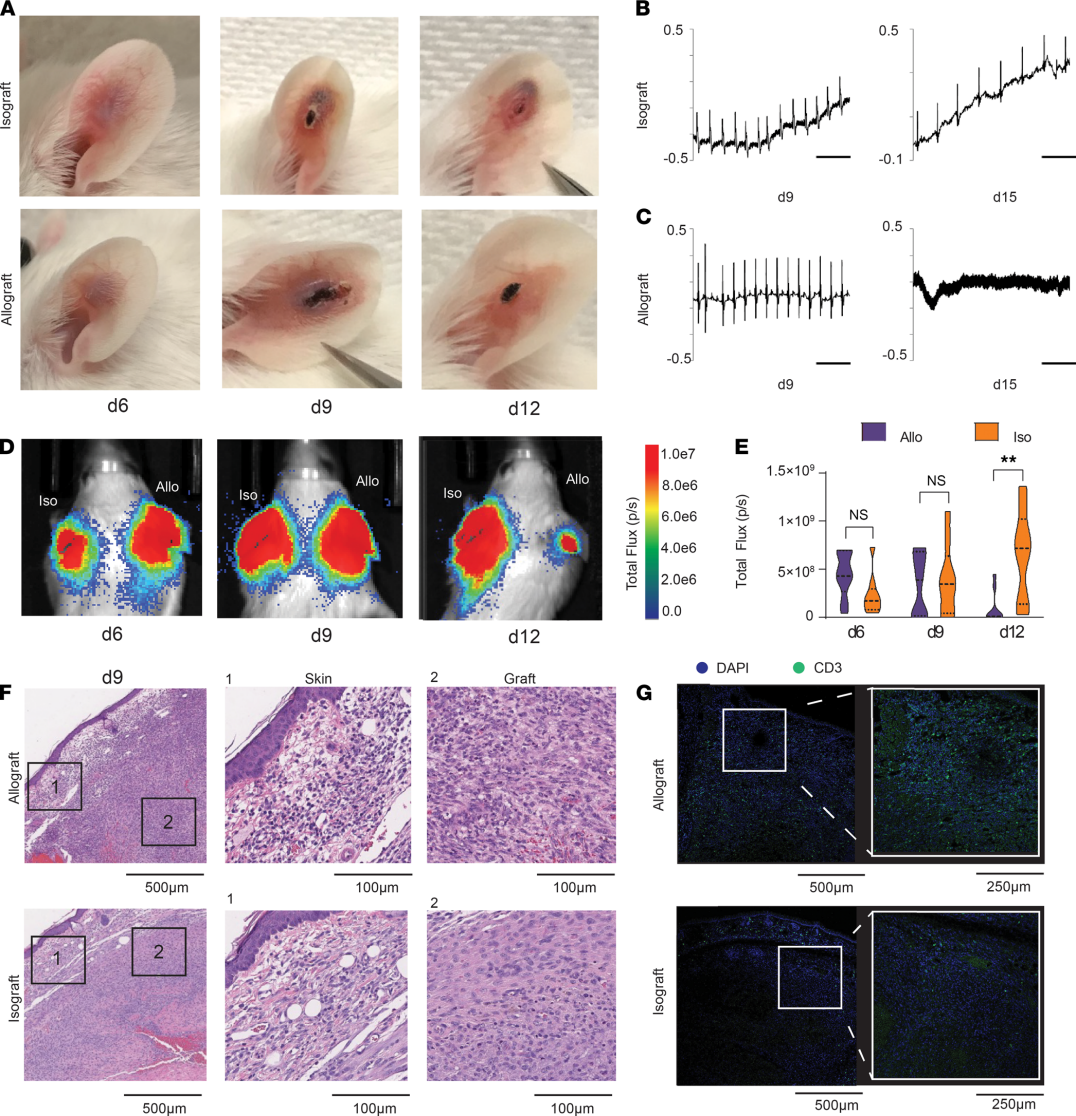
T cell infiltration precedes loss in allograft viability in organ transplant recipients. (**A**) Photographs of transplanted cardiac ear pinna iso (top) and allografts (bottom) at days 6 (d6), d9, and d12 after transplantation. (**B** and **C**) Electrocardiogram (ECG) of transplanted heart measured from electrodes placed on edge of iso (**B**) and allograft (**C**). Scale bars: 2 seconds. (**D**) Bioluminescent images depicting graft viability at d6, d9, and d12 after transplantation in a WT BALB/c mouse. Left ear; BALB/c cardiac isograft expressing firefly luciferase (FL). Right ear; C57BL/6 cardiac allograft expressing FL. Scale bar represents total flux (photon /second); red, high; blue, low. Images are representative of 9 dual graft recipient mice from 2 independent experiments. (**E**) Violin plot of total flux measured from cardiac allografts (*n =* 9) and isografts (*n =* 9) at each respective time point indicated on *x* axis. Purple, allograft (Allo); Orange, isograft (Iso). Dashed line indicates mean. **P = 0.0028 calculated by Mann-Whitney *U* test. (**F**) Images of H&E staining of allograft (top) and isograft (bottom) on d9 after transplantation. Inset box 1, skin surface region; mononuclear cell infiltration was shown in both allo- and isograft surface. Inset box 2, graft parenchyma. Massive mononuclear cell infiltration was shown in allograft parenchyma. (**G**) Images of immunofluorescent staining for CD3 (Green) and DAPI (Blue). Left; low magnification, right; high magnification of inset region. CD3^+^ T cells infiltrating allograft parenchyma.

To characterize OX40 as a biomarker in organ transplant rejection, we established a dual allograft and isograft heart transplant model. C57BL/6 (allogeneic) and BALB/c (syngeneic) hearts were harvested from 0- to 2-day-old neonatal mice and transplanted into bilateral ear pinnas of adult BALB/c mice (C57BL/6 into the right and BALB/c into the left ear pinna). Isografts appeared as a pink flat tissue after recovering from initial swelling around day 12 (d12; Figure 1A, top) and started beating 3–4 weeks later (Supplemental Video 1; supplemental material available online with this article; https://doi.org/10.1172/jci.insight.145360DS1). The appearances of allografts were similar to those of isografts by d9 (Figure 1A bottom). However, the allografts appeared white and firm after recovery from initial inflammation (d12). Allografts eventually disappeared around d30, and function was not observed. To confirm graft viability, we measured electrocardiogram (ECG) activity on ear pinna on d9 and d15. Transplanted isografts showed electrical activity at both time points (Figure 1B). The allografts demonstrated sustained electrical activity on d9, though this was lost by d15 (Figure 1C). These data collectively suggest that rejection occurred between d9 and d15, although the exact onset of rejection was not clear because of the identical macroscopic appearances between allo- and isografts at the early time points.

To determine the critical time point for graft rejection, we visualized graft viability by bioluminescent imaging (BLI). Both allo- and isodonor hearts were obtained from luciferase transgenic mice and grafted on bilateral ears (Figure 1, D and E). Allo- and isografts showed similar luciferase activity on d6, and signals were sustained at the same level until d9. Then, signaling from allografts rapidly diminished on d12, whereas that from isografts did not change, suggesting tissue destruction occurred between d9 and d12 in our model.

### T cell infiltration precedes allograft viability loss in organ transplant recipients.

We next assessed tissue damage and cell infiltration with sequential histopathological examination during this 6- to 12-day window. On d6, mononuclear cells could be seen infiltrating in s.c. tissues surrounding the graft surface, to similar extents in both allo- and isografts (Supplemental Figure 1, upper panel). This was likely due to inflammation caused by surgical invasion. At this stage, cell infiltration inside the graft was not observed in either allo- or isografts. On d9, while cell infiltration on the surface area was still similarly observed in allo- and isografts (Figure 1F, box 1), cell infiltration inside the graft was significantly increased in the allo- compared with isograft (Figure 1F, box 2). To identify if these were T cells migrating into the graft, we performed CD3 immunofluorescent staining on d9. Though CD3^+^ cell migration was limited to the surrounding area of the isograft, CD3^+^ cells could be observed entirely throughout the allograft (Figure 1G). On d12, isografts showed reduced cell infiltration, while allografts showed further increased cell infiltration (Supplemental Figure 1, lower panel). Consistent with graft viability shown in BLI, allograft tissues were overwhelmed by infiltrating cells, and cardiomyocytes were hardly detected on d12. Taken together, we hypothesized that the crucial time point to diagnose rejection is d9, when T cell migration starts to be detected but allograft viability remains.

### OX40 identifies an alloreactive CD4^+^ T cell subset infiltrating the graft.

**Figure 2:**
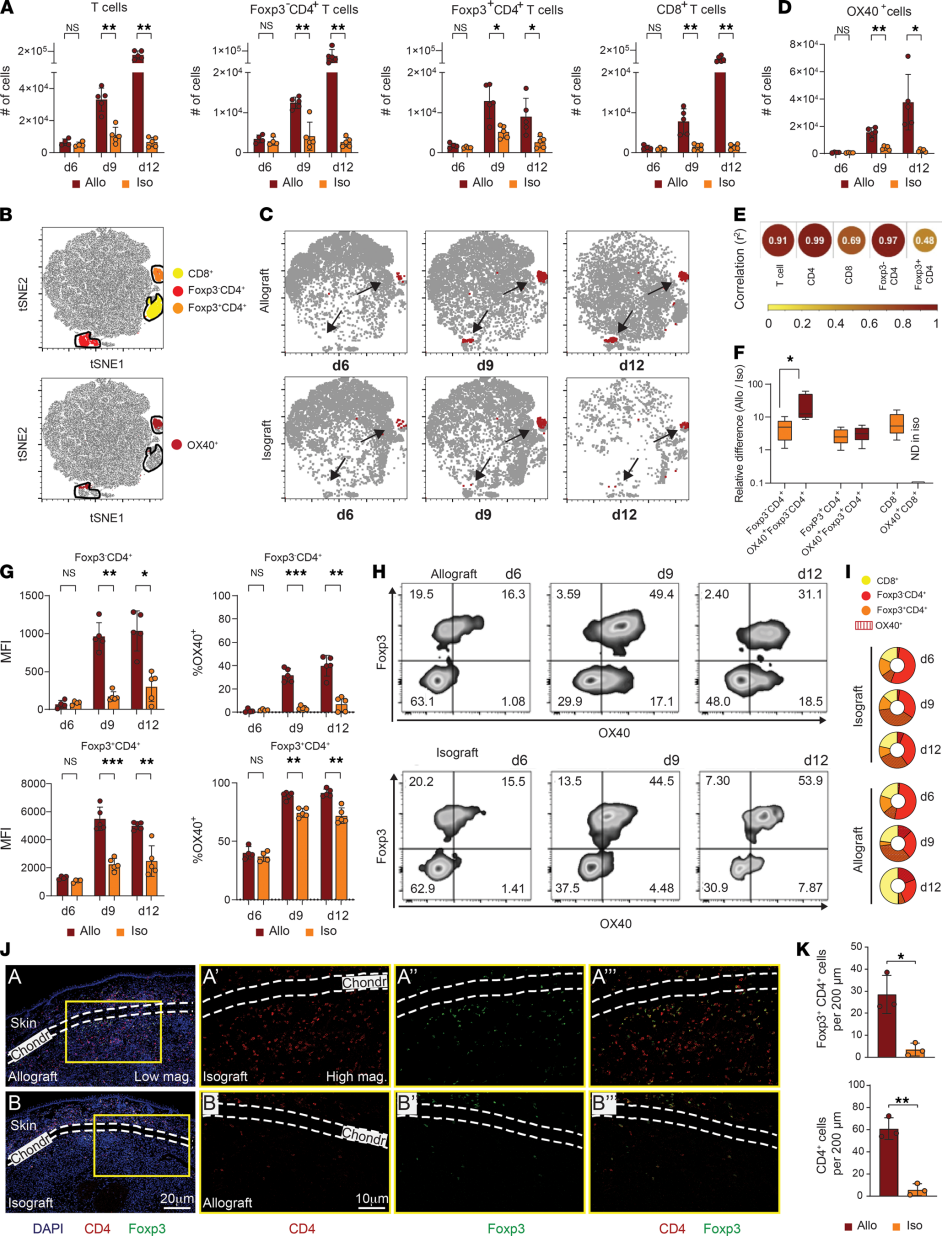
OX40 identifies an alloreactive CD4^+^ T cell subset in the graft and draining lymph node. (**A**) Quantification of T cell subsets in allo- or isografts at d6 (*n =* 4), d9 (*n =* 5) ,and d12 (*n =* 5) after transplantation. (**B**) t-SNE representations of concatenated single-cell cytometry data from allo- and isografts across all time points. Top, colors/clusters represent distribution of indicated cell phenotype. Bottom,distribution of OX40-expressing cells. (**C**) Subset t-SNE plots for allo- or isografts at d6, d9, or d12. Red indicates OX40-expressing cells. Arrows indicate 2 primary clusters associated with OX40 expression. (**D**) Quantification of the number of OX40^+^ cells. (**E**) Correlation of OX40 with T cell phenotype. Color and circle size provide a graphical representation of *r^2^* correlation value. (**F**) Relative difference in the number of cells of each T cell phenotype between allograft and isograft on d9. The *y* axis represents fold change (Allo/Iso). (**G**) Fluorescent intensity (MFI) and frequency of OX40 expression on principle CD4 T cell subsets in allo- or isografts over time. (**H**) Representative biaxial cytometry plots of OX40 versus Foxp3 expression in allo- or isografts. (**I**) The proportion of Foxp3^–^CD4^+^ (red), Foxp3^+^CD4^+^ (orange), and CD8^+^ T cell (yellow) phenotype among total graft-infiltrating T cells. Sections with shaded lines represents OX40^+^ cell frequency in each population. Each compartment represents average proportion of *n =* 4–5 samples for indicated time point. (**J**) Immunofluorescent microscopy images depicting infiltration of CD4^+^ T cells (red) and Foxp3^+^ cells (green) in areas surrounding and within allo- and isografts at d9. High-magnification images are of indicated insets. (**K**) Quantification of Foxp3^+^CD4^+^ (top) and total CD4^+^ (bottom) cell count within grafts per 200μm^2^ (*n =* 3) on d9. *P < 0.05, **P < 0.01, ***P < 0.001 calculated with Mann-Whitney *U* test between allograft and isograft. All data are representative of at least 2 independent experiments.

To determine the phenotype of the graft-infiltrating T cells, both allo- and isografts were removed on d6 (before rejection), d9 (early rejection), and d12 (late rejection), and single-cell suspensions were run by flow cytometry (Figure 2A). T cells were hardly detectable on d6 in both allo- and isografts. Consistent with histopathology, T cells in allografts progressively increased thereafter (5-fold increase from d6 to d9, and another 5-fold increase from d9 to d12), whereas T cells in isografts remained in the same range until d12. As a result, the number of T cells in allografts were 1.2- to 6.7-fold higher on d9 and 12.6- to 36.5-fold higher on d12, compared with the isografts. The numbers of 3 different T cell phenotypes — Foxp3^–^CD4^+^, Foxp3^+^CD4^+^, and CD8^+^ T cells — were characterized and also showed similar trends (Figure 2A and Supplemental Figure 2). CD4^+^ T cells were the dominant phenotype in both allo- and isografts on d6 and on d9, though CD8^+^ T cells became more dominant in the late phase of rejection (d12) in the allograft (Figure 2I).

To assess which cell phenotypes were expressing OX40, we performed T–distributed stochastic neighbor embedding (t-SNE) analysis on flow cytometry data from the allo- and isografts. OX40 expression was highly restricted to CD4^+^ T cell clusters (including Foxp3^–^ and Foxp3^+^) and was not expressed on other cell phenotypes (Figure 2B). On d6, OX40 was expressed only on Foxp3^+^CD4^+^ cells in both allo- and isografts. On d9 and d12, however, OX40 expression appeared on Foxp3^–^CD4^+^ cells in allografts, whereas this phenotype was hardly observed in the isografts (Figure 2C). The number of OX40^+^ cells demonstrated similar kinetics to that of the total T cell population (Figure 2D). The number of OX40^+^ cells in allografts was 1.8- to 7.0-fold higher on d9 and 10- to 24-fold higher on d12, compared with that in the isografts. To further address phenotypic correlates of OX40^+^ cell dynamics, we calculated Pearson’s correlation coefficients between the number of OX40^+^ cells and that of each T cell phenotype (Figure 2E). The strongest correlation was CD4^+^ T cells, specifically the Foxp3^–^CD4^+^ T cell phenotype. Importantly, because of the lack of OX40 expression on Foxp3^–^CD4^+^cells in isografts (Figure 2, G and H), the relative difference of OX40^+^Foxp3^–^CD4^+^ cells between allografts and isografts was significantly higher compared with the difference of the whole Foxp3^–^CD4^+^ phenotype (Figure 2F; 26.1 ± 23.1 fold versus 4.8 ± 3.4 fold, *P =* 0.0159).

The other phenotype observed to express OX40 was Foxp3^+^CD4^+^ T cells. Interestingly, compared with stable OX40 expression over time on Foxp3^+^CD4^+^ cells in isografts, OX40 expression on Foxp3^+^CD4^+^ cells in allografts significantly increased to nearly 100% on d9 and was sustained on d12 (Figure 2, G and H). In addition to the high OX40 expression level, Foxp3^+^CD4^+^ cells in allografts showed migration into cardiomyocytes, while Foxp3^+^CD4^+^ cells in isografts were localized to the area surrounding the graft (Figure 2, J and K). Taken together, our data suggest OX40 is a specific marker of alloreactive CD4^+^ T cells in this model of organ transplant rejection.

### OX40 identifies an alloantigen primed CD4 T cell subsets in the graft draining lymph node.

Cervical lymph node of allograft draining side (allo-draining lymph node [allo dLN]) and isograft draining side (iso dLN) were obtained and analyzed by flow cytometry. Similar to the flow cytometry results from the graft-infiltrating cells, OX40 expression was shown to be restricted to CD4^+^ T cells (data not shown). The number of T cells and OX40^+^ cells was significantly higher in the allo dLN at d9 and d12, compared with those in the iso dLN (Supplemental Figure 3A). OX40 expression levels on Foxp3^–^CD4^+^ cells were also significantly higher in the allo dLN than that in the iso dLN on d9 (*P =* 0.0079), though Foxp3^+^CD4^+^ cells did not show significant differences in OX40 expression levels between allo and iso dLN on d9 (*P =* 0.151; Supplemental Figure 3, B and C). The data collectively suggest that OX40^+^ cells are specifically identified not only in rejection site, but also in the regional lymph nodes.

### Imaging OX40 T cells detects allograft rejection prior to organ viability loss.

**Figure 3:**
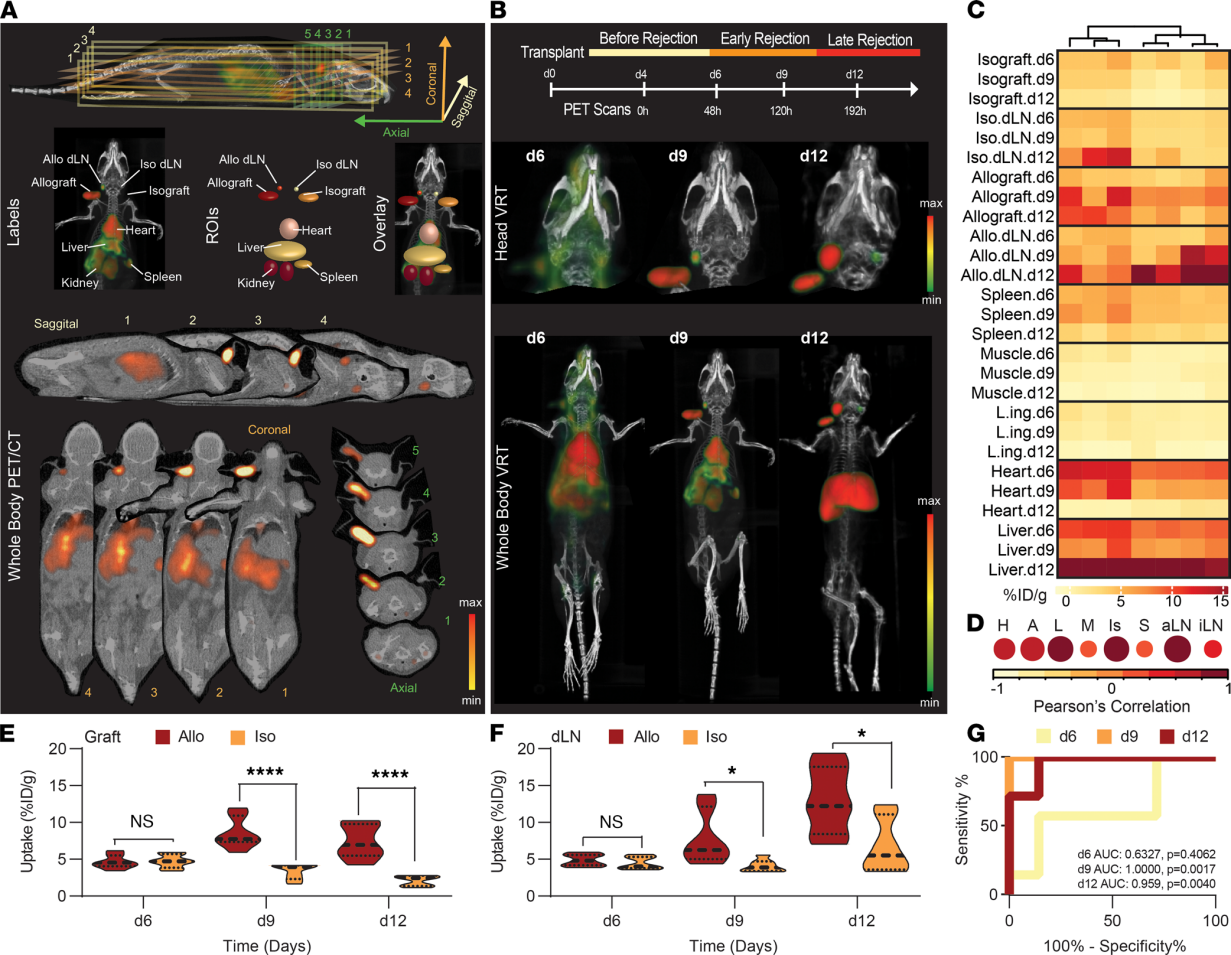
Imaging OX40 T cells detects allograft rejection prior to organ failure. (**A**) An image atlas and guide for interpreting OX40 PET/CT scans of BALB/c mice bearing ear pinna cardiac grafts from C57BL/6 (allo, right ear) or BALB/c (iso, left ear). Three-dimensional images were reconstructed, and regions of interest (ROIs) were drawn as indicated utilizing coronal, axial, and sagittal cross sections as anatomical guides. (**B**) Top: imaging schedule for transplant recipients. Middle: zoomed-in view of 3D volume rendered images (VRT) of a representative mouse bearing cardiac allografts (right ear) and isografts (left ear) at d6, d9, and d12 after transplantation. Scale bar represents OX40 PET signal intensity. Bottom: whole body images of the same respective mouse at various time points. Images are representative of *n =* 7 mice from 2 independent experiments. (**C**) Heatmap visualization of normalized max % injected dose per gram (%ID/g) OX40 PET tracer uptake values in each ROI at d6, d9 and d12 (rows) from transplant recipients (columns). (**D**) Correlation of %ID/g at each ROI versus ex vivo measurements of the same respective organs utilizing a gamma counter. Color and circle size provide a graphical representation of Pearson’s correlation value. H, native heart; A, allograft; L, liver; M, muscle; Is, isograft; S, spleen; aLN, allo dLN; iLN, iso dLN. (**E** and **F**) Comparison of %ID/g uptake calculated from image ROIs for allo- and isografts (**E**) or allo- and iso-dLNs (**F**) at d6, 9 and 12. Significant increase in uptake is shown in allograft and allo-dLN on d9 and d12. (**G**) Receiver operator characteristic (ROC) curve depicting specificity and sensitivity of OX40 PET signal-based determination of engraftment or rejection of transplant at various time points. Predictor ([graft %ID/g]^2^ + [dLN %ID/g]^2^); *n =* 14. *P < 0.05, ****P < 0.0001. Calculated with unpaired 2-tailed Student’s *t* test.

To noninvasively assess the alloreactive T cell population identified by OX40 expression during graft rejection, we developed a ^89^Zr radiolabeled ImmunoPET probe targeting OX40. Mice (*n =* 7) bearing dual cardiac iso- and allografts, in their left and right ear pinna, respectively, were imaged longitudinally on d6 (before rejection), d9 (early rejection), and d12 (late rejection; Figure 3, A and B). OX40 ImmunoPET imaging and quantification recapitulated the time course kinetics of alloreactive T cell expansion and infiltration observed during our flow cytometry studies (Figure 3, E and F). Specifically, OX40 images revealed progressive signal increases in the allo dLN that was not observed in the iso dLN (d6: allo dLN 4.88 ± 0.31 %ID/g versus iso dLN 4.491 ± 0.33 %ID/g; d9: allo dLN 7.87 ± 1.38 %ID/g versus iso dLN 4.10 ± 0.32 %ID/g, *P* < 0.05; d12: allo dLN 13.10 ± 1.73 %ID/g versus iso dLN 6.78 ± 1.39 %ID/g, *P* < 0.05). Correspondingly, the allografts showed marked imaging signal increase over its isograft-counterpart overtime (d6: allograft 4.6 ± 0.4 %ID/g versus isograft 4.7 ± 0.4 %ID/g; d9: allograft 8.5 ± 0.8 %ID/g versus isograft 3.4 ± 0.4 %ID/g, *P* < 0.0001; d12: allograft 7.3 ± 0.9 %ID/g versus isograft 2.1 ± 0.3 %ID/g, *P* < 0.0001). Whole body images revealed that the allo-driven immune response was localized (Figure 3C). Signal enhancement at secondary lymphoid sites or non-dLNs was not observed. Imaging signals observed at other sites in the body, such as the heart and liver, were associated with imaging probe clearance from the blood over time as expected for a full length antibody (27, 28). Ex vivo analysis of isolated tissues confirmed the accuracy of our image-based observations, as indicated by Pearson’s correlation coefficients between the ex vivo biodistribution measured tracer uptake (%ID/g) and the in vivo region of interest (ROI) measured tracer uptake (%ID/g; Figure 3D). Notably, this imaging approach could be utilized to diagnose graft rejection (allograft versus isograft) with high sensitivity and specificity at the early rejection time point (d9; AUC, 1.0; *P =* 0.0017; *n =* 14; Figure 3G).

### The clinical immunosuppressant tacrolimus reduces OX40 alloreactive T cell numbers and prevents allograft rejection.

**Figure 4:**
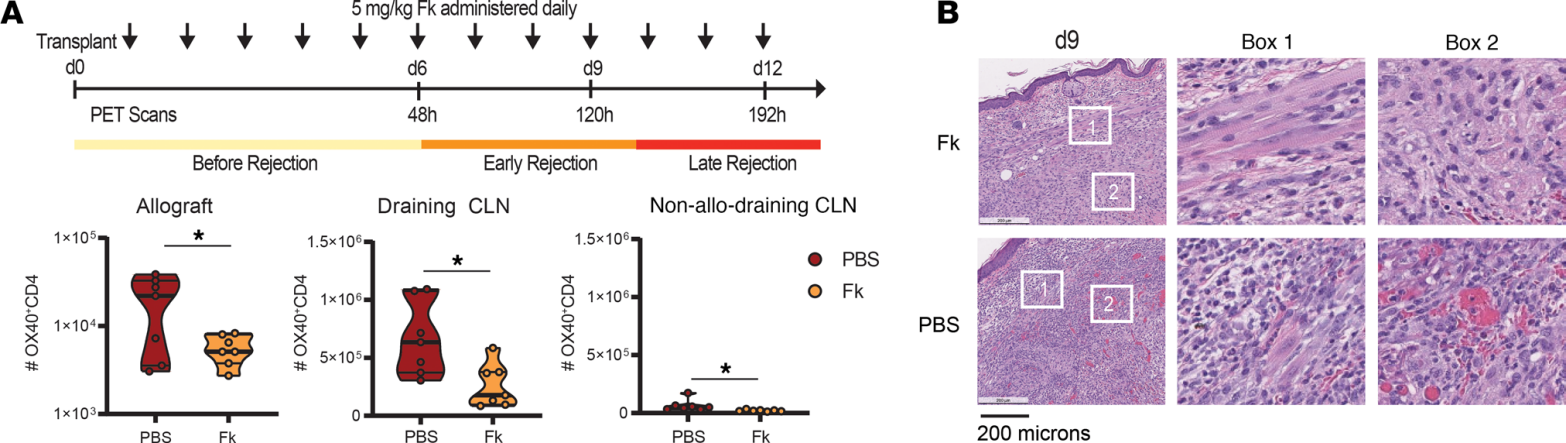
The clinical immunosuppressant tacrolimus reduces OX40 alloreactive T cell numbers and prevents allograft rejection. (**A**) Top: tacrolimus (Fk) immunosuppressant treatment schedule following cardiac ear pinna allograft transplantation. Bottom: quantification of the number of OX40^+^ CD4 T cells in the allograft, allo-draining, and non-allo-draining cervical lymph node (CLN) for allograft recipients treated with immunosuppressant (Fk) or vehicle (PBS) at d9 after transplantation. (**B**) Images of H&E staining depicting cellular infiltrate in allografts of immunosuppressed (Fk) or vehicle-treated mice (PBS) at d9. Boxes 1 and 2 are zoomed-in regions of the indicated insets. Box 1, graft surface; box 2, graft parenchyma.

To test if clinical immunosuppressive regiments had an effect on OX40 T cell numbers, we utilized tacrolimus (drug abbreviation, Fk), a calcineurin inhibitor commonly used in the clinic after allogeneic organ transplantation to prevent organ rejection. Tacrolimus was given to the mice at a dose of 5 mg/kg daily, and the OX40 infiltration into the allograft was measured. Tacrolimus led to significantly decreased numbers of OX40^+^CD4^+^ T cells in the allograft (Fk 5638 ± 783 versus PBS 19,221 ± 5519, *P* < 0.05), and allo dLN (Fk 260,251 ± 18,752 versus PBS 665,893 ± 120,481, *P* < 0.05), during the period of immunosuppression (Figure 4A). Histology confirmed reduced immune infiltrate and reduced signs of graft injury in tacrolimus-treated compared with vehicle control mice (Figure 4B).

### OX40 imaging enables specific and sensitive measurement of immunosuppression in allograft recipients.

**Figure 5:**
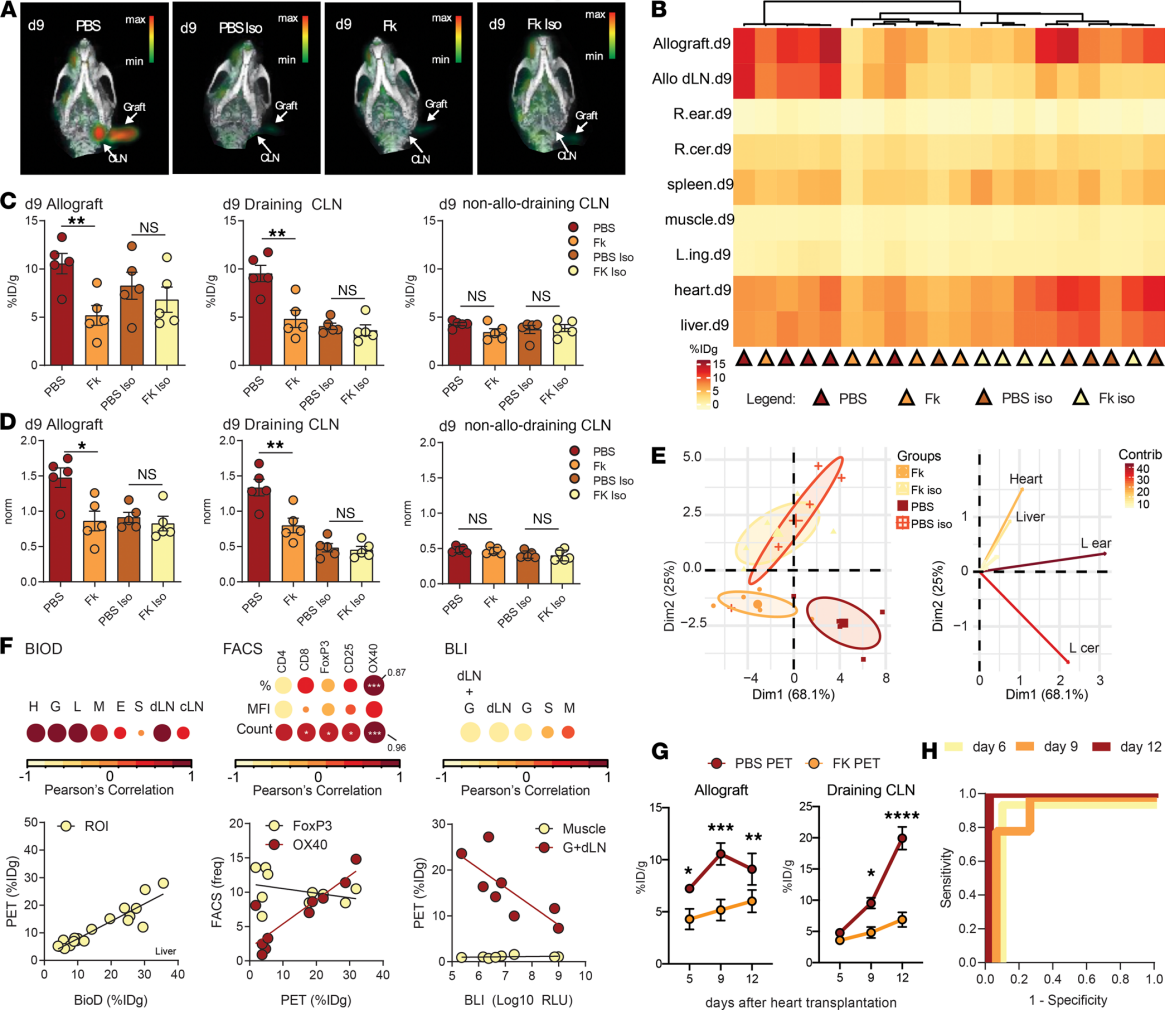
OX40 imaging enables specific and sensitive measurement of immunosuppression in allograft recipients. (**A**) Representative OX40 or isotype (Iso) control PET/CT images of vehicle-treated (PBS) or immunosuppressed (Fk) mice bearing a single allograft (left ear) at d9 after transplantation; *n =* 5 per group. Data are from 2 independent experiments. CLN, cervical lymph node. (**B**) Heatmap of max %ID/g in regions of interest (rows) in allograft recipients (columns) from PBS or Fk cohorts at d9. (**C** and **D**) Comparison of %ID/g (**C**) or %ID/g normalized (**D**) by blood background value in the allograft, allo-draining CLN, and non-allo-draining CLN between PBS and fk groups at d9. (**E**) Principal component analysis identifies Fk-treated mice from PBS mice. Each dot represents an individual mouse (left). Each line represents key drivers of the delineation (right). L ear, left ear (allograft); L cer, left CLN. (**F**) Correlation of %ID/g in each ROI versus: left-top, %ID/g of the same respective organs measured ex vivo by a gamma counter; left-bottom, example plot for Liver ROI; middle-top, number of each cell population in allo-CLN; middle-bottom, example plot depicting %ID/g correlation with target OX40 and nontarget FoxP3 frequency measured via FACS; right-top, allograft bioluminescent signals; and right-bottom, example of the correlation of PET Graft + all-CLN ROI values with BLI signal, and PET muscle ROI values with BLI signal. Color and circle size provide a graphical representation of Pearson’s correlation value. (**G**) Time course plots of average %ID/g for PBS or Fk mice in allograft (left) and in draining CLN (right). (**H**) ROC curve depicting specificity and sensitivity for determination of PBS or Fk mouse at various time points by the sum of %ID/g values from allograft and allo-CLN; *n =* 10. * *P* < 0.05, ** *P* < 0.01, *** *P* < 0.001, **** *P* < 0.0001. Calculated with unpaired Student’s *t* test.

Finally, to assess whether OX40 imaging could aid in the management of immunosuppression for subjects receiving allografts, we performed OX40 imaging studies in the tacrolimus treatment mouse model. Allograft recipients that received either tacrolimus (*n =* 5) or vehicle control (*n =* 5) were imaged with our OX40 PET probe or an isotype PET probe (*n =* 5/group) as an additional control. The same imaging schedule and procedure were followed as our established dual allograft/isograft model. We focused our analysis on d9 after transplantation, as this time point coincided with subclinical signs of graft rejection such as T cell infiltration, while the grafts did not show macroscopic signs of clinical rejection and, importantly, were still viable. Imaging revealed that, at d9, transplant recipients receiving vehicle treatment had high OX40 PET signals in the allograft and allo dLN compared with that in subjects that were being immunosuppressed with tacrolimus (Figure 5A). Quantification of the OX40 PET signal from ROIs identified the alloreactive immune response to be locally driven, while the PET tracer showed clearance from the blood via the liver (Figure 5B). Statistically significant differences were observed in OX40 PET signal between tacrolimus immunosuppressed recipients and vehicle-treated controls in the allograft (*P* < 0.01), the allo dLN (*P* < 0.01), but not in the non-dLN (*P =* ns), where all signals were at background level (Figure 5C). This PET signals were specific for OX40, as the isotype PET signal did not recapitulate these differences in these ROIs. Of note, the isotype scans did show higher background in the allograft itself. This might be due to a longer circulation time of isotype tracer than the whole antibody tracer (27). Thereby, when the scans were normalized for the nonspecific signal coming from the blood, the background difference between OX40 and isotype was not observed (Figure 5D). We performed principal component analysis and confirmed that the PET signals coming from the allograft and allo dLN were in fact key drivers of the delineation seen in the images between the tacrolimus and vehicle control groups (Figure 5E). PET ROIs correlated well with ex vivo measurements via BioD (Figure 5F). Furthermore, PET measurements correlated with FACS measurements of OX40 expression, as well as the extent of allograft rejection measured via BLI (Figure 5F). Plots of OX40 PET scans show that the difference between the groups was statistically significant from the early time points (Figure 5G). Finally, OX40 PET imaging was able to perfectly delineate mice which were immunosuppressed versus those that were not (d6 AUC = 1, *P* < 0.01; Figure 5H). All of these data suggest that OX40 PET is capable of monitoring allograft transplant recipient immunosuppression.

## Discussion

This work demonstrates, for the first time to our knowledge, the ability of OX40 immunoPET to noninvasively monitor alloreactive T cell dynamics during organ transplant rejection. In an ear pinna cardiac graft model of rejection, OX40 immunoPET signals identified allograft rejection at early time points (d9) preceding drastic T cell proliferation, infiltration, and destruction of the graft. OX40 immunoPET detected signs of alloreactive T cell–mediated rejection prior to imaging methods monitoring graft viability loss. OX40 immunoPET also clearly distinguished alloreactive T cells in the allo dLN compared with the iso dLN or other secondary lymphoid sites. This is an advantage to other T cell imaging approaches that are nonspecific for activated T cells. OX40 immunoPET was capable of measuring immune activation and the subsequent drop in OX40 PET signals induced by immunosuppression with the clinically approved drug tacrolimus. The degree of immunosuppression as indicated by decreased OX40 immunoPET signals correlated with improved graft viability. Taken together, this proof-of-concept study demonstrates that OX40 immunoPET merits further investigation as a tool to detect graft rejection and guide appropriate patient treatment.

In our previous OX40 imaging work in a cancer vaccine model (26), we utilized a ^64^Cu (half-life, 12.7 hours) OX40 ImmunoPET tracer. Our newly developed ^89^Zr-labeled OX40 immunoPET radiotracer described here enabled true longitudinal monitoring, due to the extended half-life of the ^89^Zr radioisotope (half-life, 78.4 hours). In this study, longitudinal observation enabled the comparison of OX40 immunoPET with other modalities (BLI and FACS) and the ability to capture dynamic T cell activation over time.

In this study, we chose to use an ear pinna heart transplant model to study and characterize OX40 as a surrogate biomarker of alloreactive T cells. The ear pinna heart transplant model, which was developed more than 4 decades ago (29, 30), has been widely used across many experimental settings to assess recipient immunological reactions (31, 32) or to prove establishment of immunological tolerance (33, 34), although this is the first report to our knowledge of PET imaging research to utilize this model. The advantage of this model for imaging study is that it enables evaluation of immune response to allo- and isografts, as well as their respective dLNs, simultaneously in the same animal. Since one of the aims of this study was to determine whether immunoPET could distinguish immunological rejection from nonimmunological inflammation, the isografts provided the perfect internal control. In contrast, the widely used heterotopic heart transplantation model would require the preparation of another animal to study the isograft control. The heterotopic heart transplantation model has challenges, including variation among each animal in terms of postoperative situations, such as bleeding, dehydration, and quality of anastomosis. The dual–heart transplantation model enabled us to account for these variables, leaving observed differences in allograft versus isograft to be attributed to immune reaction against alloantigens.

There are several limitations to the ear pinna heart transplantation model for the study of rejection. First, diagnosis of rejection for ear pinna heart grafts is more complicated compared with that of fully vascularized heart grafts because of lack of initial heart beating. To address the critical time frame for rejection, we implemented BLI, which had been previously used for tracing viability of transplanted heart tissues (19, 35, 36). According to the reduction of photons from viable cardiac cells expressing the luciferase reporter gene, we were able to determine that graft survival time was around 12 days. This survival time was consistent with other reported heart transplantation models. Interestingly, despite aggressive T cell infiltration observed in allograft by immunofluorescence staining and flow cytometry, BLI signals between allo- and isografts were still comparable on d9. We designated this time point as early rejection, as it corresponded clinically to the subclinical rejection phase when we can only diagnose rejection by biopsy.

Another caveat of using the ear pinna heart transplant model for studying rejection is the difference in theoretical tracks of lymphocyte infiltration compared with heterotopic heart transplants. In contrast to cell infiltration of vascularized heart grafts from microcirculation, those cells found in the ear pinna heart could migrate directly from skin (37). It has been reported that skin tissue has abundant Tregs to maintain tolerance for commensal microbes (38–40). It is known that mouse Treg cells constitutively express OX40 (41, 42). Consistent with that, our data show a high expression of OX40 on Foxp3^+^CD4^+^ T cells. We confirmed via immunofluorescence staining that Foxp3^+^CD4^+^ cells were localized in the graft surface area that is attached to the skin lesion in the isograft. In the allograft, the expression level of OX40 was higher than isograft, and Foxp3^+^ cells migrated into the graft parenchyma. While this skin-resident migrating OX40^+^Foxp3^+^CD4^+^ Treg population is presented as a potential confounder in this preclinical model, we do not expect these regulatory cells to play a role in the human organ transplant setting. Furthermore, it should also be noted that, unlike mice, human Tregs do not constitutively express OX40 (43). Because of those limitations, further preclinical studies should be performed before clinical trials by using physiological transplantation models such as murine kidney transplantation.

In this study, we focused on OX40 as an imaging biomarker of transplant rejection. The presence of T cell migration in the graft is the strongest clinical evidence for T cell–mediated rejection. We did not pursue general markers of T cell phenotype, such as CD3, CD4, or CD8, because these targets are constitutively expressed, making it more difficult to quantify their changes during a rejection episode. We sought to select a target that was highly restricted to alloreactive T cells in the rejection setting. OX40 is a TNF family member that serves as a T cell costimulatory molecule. Unlike other costimulatory molecules, however, OX40 is not expressed on resting T cells (44, 45). It has been reported that T cells express OX40 24 hours to 4–5 days after initial antigen encounter, a suitable time window for imaging rejection events. Signals from OX40-OX40L ligation promote clonal expansion, prolong survival of proliferating T cells, and generate memory in antigen-specific T cells (46, 47). OX40 is also expressed on the memory CD4^+^ T cell phenotype that do not express other costimulatory molecules (48). It has been previously shown that OX40^+^CD4^+^ T cells are fundamental for T cell migration and rejection of allografts. OX40-OX40L signal blockade reduces migration of CD4^+^ T cells into the allograft, as well as the dLN, and prevents skin allograft rejection mediated by naive or memory CD4^+^ T cell transfer (48–50). In agreement with this data, our current study shows that OX40 expression was rarely expressed on CD4^+^ conventional T cells (Tcons) at baseline when rejection had not been initiated, and it increased accordingly with the progression of rejection. Importantly, since CD4^+^ Tcons migrating into isograft did not express OX40, the contrast between allografts and isografts in terms of the number of OX40^+^CD4^+^ T cells was significantly higher than that of whole CD4^+^ T cells. This data confirm that OX40 is a specific marker of alloreactive T cells that was more sensitive than assessing CD4^+^ T cells alone.

As we have already alluded to, in addition to Tcons, we also observed Foxp3^+^ Tregs that were expressing high levels of OX40. Although there is no doubt that murine Foxp3^+^ Tregs have antiinflammatory effects, increase in OX40 expression on Tregs is not a sign for reduced inflammation. Indeed, in contrast to its role on Tcons, OX40 signaling negatively impacts Foxp3^+^ T cells. Vu et al. have shown that OX40 stimulation abrogates suppressive function of Foxp3^+^cells in vitro (51). Kinnear et al. also reported that OX40 signal blockade generates stronger suppressive capacity on Tregs in vivo in a skin transplant model (48). Those data are in line with our observations here and collectively suggest that OX40, regardless of its expression on Foxp3^–^ or Foxp3^+^ T cells, is contributing to transplant rejection.

Our data show that OX40 expression was found mainly on CD4^+^ T cells, but not on CD8^+^ T cells. Previous reports suggest that CD8^+^ T cells express OX40 at lower levels and later time points after in vitro stimulation compared with CD4^+^ T cells (52, 53). In the current study, although the level of expression was quite low, CD8^+^ T cells showed a gradual increase in OX40 fluorescence intensity according to rejection progress. Since we based the OX40 fluorescence expression cut-off according to the intensity of isotype control, it is possible that we dismissed OX40^+^CD8^+^ T cells that had low OX40 expression. Neutrophils, NK, and NKT cells can also express OX40 (47), although we did not observe this in our study. While any cell type expressing OX40 could theoretically contribute to our immunoPET signals, our data suggest the signal observed is primarily due to alloreactive CD4^+^ T cells during rejection in this model.

Regarding the role of OX40 in clinical transplantation, several small-cohort investigations have shown the relevance of OX40 to rejection in clinical kidney transplant recipients. Afaneh et al. have measured the mRNA levels of OX40 and OX40L in urinary cells obtained from kidney transplant recipients. Patients who developed acute rejection showed higher expression levels of OX40/OX40L, and these levels were correlated to reversibility of rejection and graft survival (54). Wang showed similar trends in PBMC analysis (55). Significant increases in OX40 mRNA expression in the kidney biopsy specimens of chronic T cell–mediated rejection patients was confirmed in a comprehensive analysis reported by Curci et al. (56). These preliminary reports help establish the potential clinical significance of imaging OX40^+^ T cells in the human transplantation setting.

On the other hand, many other immune cell makers have been identified as the signature of transplant rejection by gene profiling studies (57–59). The immunoPET strategy may be utilized with these other molecules. Indeed, we recently published that ICOS PET is also capable of detecting activated T cells following CAR T cell therapy (60). Recently, high-throughput sequencing analysis has provided specific molecular evidence in each rejection type (61, 62). By incorporating those specific target molecules as new tracers, ImmunoPET might be able to distinguish rejection types in the future.

Several recent studies reported that new imaging tracers targeting cell surface molecules such as Ga^68^-PSMA or Ga^68^-DOTATATE PET might improve cost effectiveness (63–65), whereas product cost themselves are relatively expensive. OX40 immunoPET might be expensive compared with standard examinations in the clinic. However, it may resolve safety issues related to biopsy for organ transplant rejection monitoring. For patients, ImmunoPET can be performed without hospitalization and may shorten the time to diagnosis. For clinicians, it provides 3-dimensional information of T cell infiltration for the whole organ that is not available from a needle biopsy. Repeated scans may enable clinicians to evaluate treatment effects, and protocol scans might replace protocol biopsies and increase the chance of identifying patients who are not responding to or adherent to their immunosuppressive regimens. Those advantages may compensate for the product costs and improve cost-effectiveness.

Though more investigation is required, a route to the clinic for OX40 immunoPET is already underway. An anti-OX40 monoclonal antibody is under investigation as an immune checkpoint cancer therapy. The first clinical application of OX40 immunoPET is planned for cancer treatment immune monitoring by using this anti-OX40 antibody. With some slight modifications to the clinical cancer imaging approach, we believe OX40 immunoPET holds great promise for the organ transplant setting.

## Methods

### Mice.

Eight- to 12-week-old BALB/c mice were purchased as heart recipients from the Jackson Laboratory. Newborns (0–48 hours old) for heart donors were obtained from timed-pregnant C57BL/6 mice purchased from the Jackson Laboratory, or luciferase gene transgenic (*luc^+^*) C57BL/6 or BALB/c mice that were bred in our animal facility. C.Cg-Foxp3^tm2Tch^/J (Foxp3^EGFP+^BALB/c) mice, purchased from the Jackson Laboratory, were bred and used for IHC experiments.

### Ear pinna heart transplantation and monitoring graft survival.

Heterotopic heart transplantation was performed as described previously (1). Briefly, newborn donors and recipients were anesthetized with isoflurane. Obtained newborn hearts were placed into ear pinna through the tunnel made from small incision on occiput skin by using 14-gauge 1” to 1/4” Jelco IV Catheter (Smiths Medical). Heart graft viability was assessed with daily visual observation. In case of *luc^+^*-donor, BLI was performed every 2 days from d0. D-luciferin (Biosynth) was injected i.p. 10 minutes prior to image acquisition with Ami Imager. Firefly luciferase images were analyzed with Aura software (Spectral Instruments Imaging).

### Flow cytometry.

Heart grafts and draining cervical LNs were removed at designated time points. Heart tissues were digested with 1 mg/mL Collagenase II (Roche) and 0.1 mg/mL DNase I (MilliporeSigma) at 37°C for 30 minutes. Single-cell suspension was incubated with FcR blocking reagent (Miltenyi Biotec) for 5 minutes at 4°C and subsequently stained with the following antibody mixture: Pacific Blue CD45.2 (catalog 104), FITC TCRβ (catalog H57-597), Brilliant Violet 711 CD4 (catalog GK1.5), and Brilliant Violet 650 CD8α (catalog 53–6.7, all purchased from BioLegend), as well as PE OX40 (catalog OX-86) or isotype (rat IgG1κ, both purchased from eBioscience). For dead cell staining, LIVE/DEAD Fixable Aqua Dead Cell Staining (Thermo Fisher Scientific) was used. Cells were fixed/permeabilized with Foxp3 staining kit (ThermoFisher), and stained with APC Foxp3 (FJK-16s, eBioscience). Data were acquired on an LSR II flow cytometer (BD Biosciences) and analyzed with FlowJo 10.6.1 (BD Biosciences). Number of cells were calculated from the count of Precision counting beads (eBioscience) according to manufacturing protocol.

### ECG recording.

Mice were anesthetized with isoflurane (2% with 100% oxygen flow) and placed in supine position on a board. ECG recordings were obtained with s.c. stainless steel needle electrodes connected by an insulated cable to a biopotential amplifier (Animal Bio Amp, ADInstruments) and an acquisition system (Powerlab 16/30, ADInstruments). Two leads were placed in the pinna of the ear to allow for local bipolar recordings of the implanted hearts. Limb lead II ECGs were also recorded for comparison. The data were sampled at 1000 Hz, filtered with a low pass of 50 Hz, and analyzed with LabChartPro 7.3.7 software (ADInstruments).

### Histopathology/IHC.

For H&E staining, graft specimen was fixed in 10% formalin and sent to HistoWiz Inc. For IHC, heart grafts in the ear pinna were harvested and fixed in 4% paraformaldehyde (in PBS, pH 7.4; Electron Microscopy Services) overnight at 4°C, embedded. and cryosectioned as 10 μm slices. Tissues were permeabilized with 0.5% TritonX-100 (in PBS) for 10 minutes at room temperature and blocked with 5% donkey serum (Abcam), 0.1% TritonX-100, 1% BSA, and 0.02% sodium azide (NaN_3_) in PBS (pH 7.4) (all from Sigma-Aldrich) for 30 minutes at room temperature, followed by incubation with primary antibodies in the same blocking solution overnight at 4°C. The following day, tissues were washed with PBS 3 times at 5-minute intervals and then incubated with secondary antibodies diluted in PBS containing 0.1% TritonX-100, 1% BSA, and 0.02% NaN_3_ for 2 hours at room temperature. After washing with PBS 3 times for 5-minute durations, tissues were mounted in anti-fade fluorescent mounting medium (DAKO) and sealed with cover slips. The following primary antibodies were used: rat anti-CD3 (1:100; ab56313, Abcam), rat anti-CD4 (1:100; BD Biosciences), and chicken anti-GFP (1:1,000; Aves Labs). Alexa Fluor secondary antibodies (488 [Invitrogen] or 647 [Jackson ImmunoResearch], 1:500) and DAPI (1:10,000; Invitrogen) were also used for visualization. Representative confocal images from sections were captured using LSM880 confocal microscope (Zeiss). Image analyses were performed using Zen Software (Zeiss) and Photoshop CS6 (Adobe Systems). Cell quantification was performed from confocal images of grafted neonatal hearts adjacent to the cartridge in the adult pinna using ImageJ (NIH).

### DFO conjugation and ^89^Zr labeling with OX40 mAb.

Detailed procedures for DFO-isothiocyanate (DFO) conjugation and ^89^Zr were done following our previous study. Briefly, 1 mg of rat anti-OX40 monoclonal antibody (Bio X Cell, clone OX86) were diluted to 1 mg/mL with PBS, adjusted to pH 8.8–9.0 with 0.1M Na_2_CO_3_, and protein concentrations were measured with NanoDrop (Thermo Fisher Scientific). DFO-SCN were dissolved in DMSO, and they were subsequently added to OX86 and incubated for 60 minutes at 37°C. DFO-SCN-OX40 mAb was purified with Vivaspin filter. Finally, OX40 mAb concentration was determined by NanoDrop. ^89^Zr oxalate was mixed with 1M Na_2_CO_3_ so that pH reaches 7.1–7.8. DFO-OX40 mAb was added to ^89^Zr oxalate and incubated for 60 minutes at 37°C. ^89^Zr-DFO-OX40mAb was then purified from free ^89^Zr oxalate using spin desalting columns. Finally, radiochemical purity was assessed by chromatography strip and Instant Thin Layer Chromatography (iTCL) (Biodex). Also, an isotype control tracer was prepared as ^89^Zr-rat IgG1 mAb to evaluate the tracer specificity to targeted lesions. Rat IgG1 was conjugated with DFO-SCN and labeled with ^89^Zr in the same manner with OX86. Single pot stoichiometric conjugation of amines in the scaffold backbone with Deferoxamine-maleimide yielded 2–3 chelates per mAb. Subsequent radiolabeling results in radiochemical products with high yield and purity (>99%).

### PET/CT imaging.

In total, 1.5–2 MBq of ^89^Zr-DFO-OX40mAb were administered i.v. to orthotopic mice, and sequential standalone PET/CT scans were acquired. Time points were selected considering the physical half-life of ^89^Zr and typical long circulation times of antibodies. PET/CT studies were performed on an Inveon rodent model PET/CT scanner (Siemens Medical Solutions USA Inc.). Anesthesia was induced with 2.5% isoflurane and maintained at 2%, and mice were placed in a prone position in the scanner. Ten minutes of attenuation correction of CT scan was performed just before the 15-minute static 3D PET scan. Image reconstructions were carried out on an Inveon Acquisition Workplace software (Siemens Preclinical Solutions) using an ordered subset expectation maximization 3D/maximum *a posteriori* reconstruction algorithm. Mice bearing dual grafts were injected with 50–100 mCi of ^89^Zr-DFO-OX86 or ^89^Zr-DFO-rat IgG1 and imaged at 48, 120, and 192 hours after injection, corresponding to d6, d9, and d12 after organ transplantation.

### Statistics.

Statistical analyses were performed using Prism 8 (GraphPad Software) and R version 3.5.1 (Comprehensive R Archive Network (CRAN) project (http://cran.us.r-project.org) with R studio version 1.1.453. *P* values for 2 group comparison was calculated by Mann–Whitney U test or two-tailed student *t* test. Heatmaps were generated using Pheatmap version 1.0.12. Principal component analysis was performed using the FactoMineR package version 1.41 and visualized using the factorextra package version 1.0.5.

### Study approval.

Animal protocols were approved by the IACUC of Stanford University.

## Author contributions

TH, ATM, and TWN conceived, designed, and conducted the experiments, with help from PYL, ZX, and FS. TU performed and analyzed IHC with AC. KS performed and analyzed ECG. JB helped establishment of murine ear pinna heart transplant model. RN and SSG helped with critical advices and discussion. TH, ATM, and TWN wrote the manuscript with contribution from all authors, and RN and SSG revised the manuscript.

## Supplementary Material

Supplemental data

Supplemental Video 1

## Version 1 07/08/2021

Electronic publication

## Version 2 07/12/2021

The grant funding information was updated.
