# Supplementary material for: Imaging alloreactive T cells provides early warning of organ transplant rejection
Source: JCI Insight. 2021 Jul 8;6(13):e145360. doi: 10.1172/jci.insight.145360 (PMC8410037; doi:10.1172/jci.insight.145360)
Supplement: Supplemental data [file jciinsight-6-145360-s006.pdf]

SUPPLEMENTAL DATA

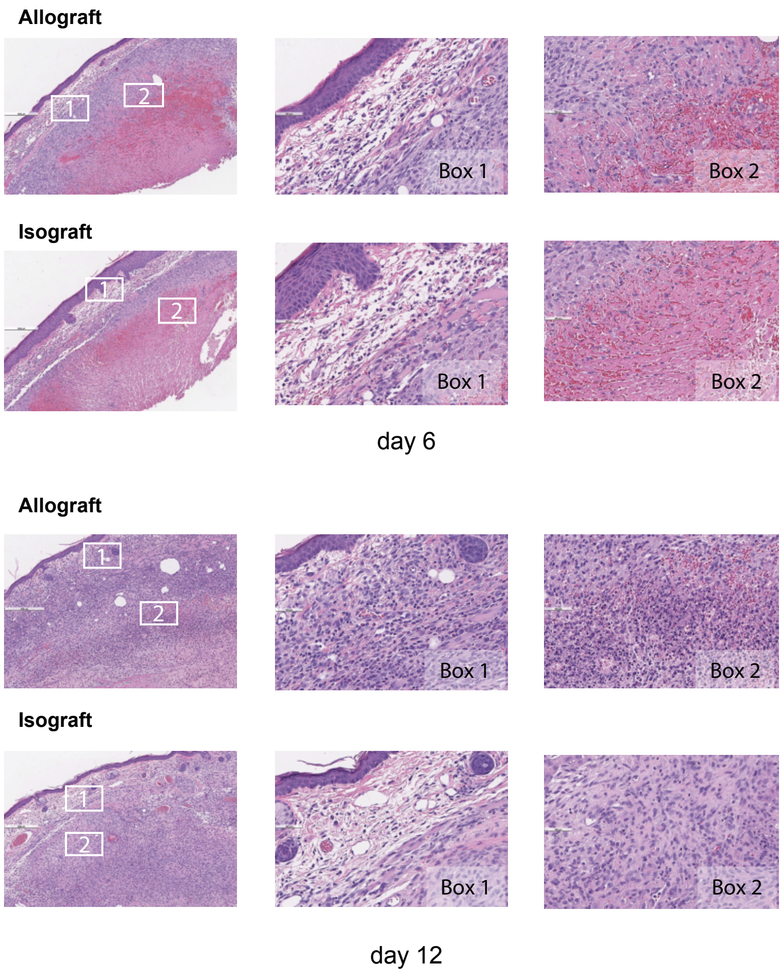

Fig. S1

HE staining of allograft and isograft. Box1 and Box2 show higher magnification in surface area and graft parenchyma, respectively. Upper panel; d6. Both allo and isograft revealed skin stromal edema around heart graft with some cell infiltration (box 1). Inside lesion of allo- and iso-graft (box 2) shows hemorrhage and few nucleusnuclei; suggests central necrosis cause by ischemia. Lower panel; d12. Allograft tissues replaced with cell infiltration in both surface (box 1) and inside graft (box 2). Isograft shows reduction in surface cell infiltration. Spindle shape cells are detectable; suggest recovery of cardiomyocytes.

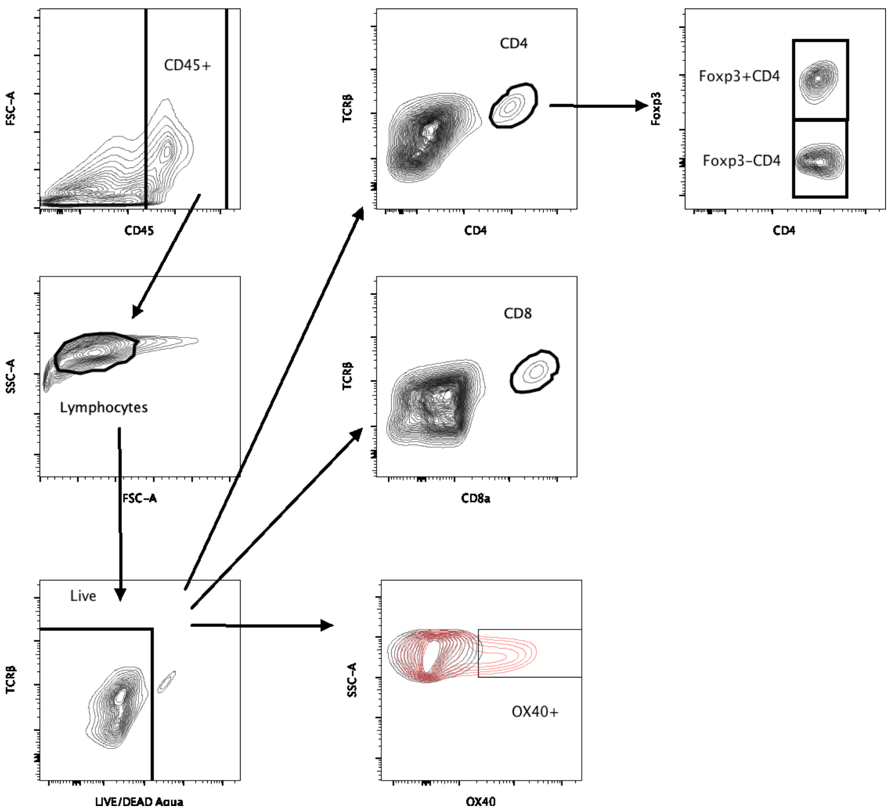

Fig. S2

Sorting strategy for graft infiltrating cells. Data was concatenated from allo and isograft at all the time points (d6, d9, d12). Pacific Blue CD45.2, FITC TCRβ, Brilliant Violet 711 CD4, Brilliant Violet 650 CD8α, APC Foxp3. Living cell gate was determined by LIVE/DEAD Fixable Aqua Dead Cell Staining. PE OX40 (OX86; red line) or isotype (rat IgG1 kappa; black line) was stained on the same panel and overlaid on singlet/CD45.2^{+}/lymphocyte/live gated cells. OX40^{+}gate was made on cells that do not overlap on isotype staining.

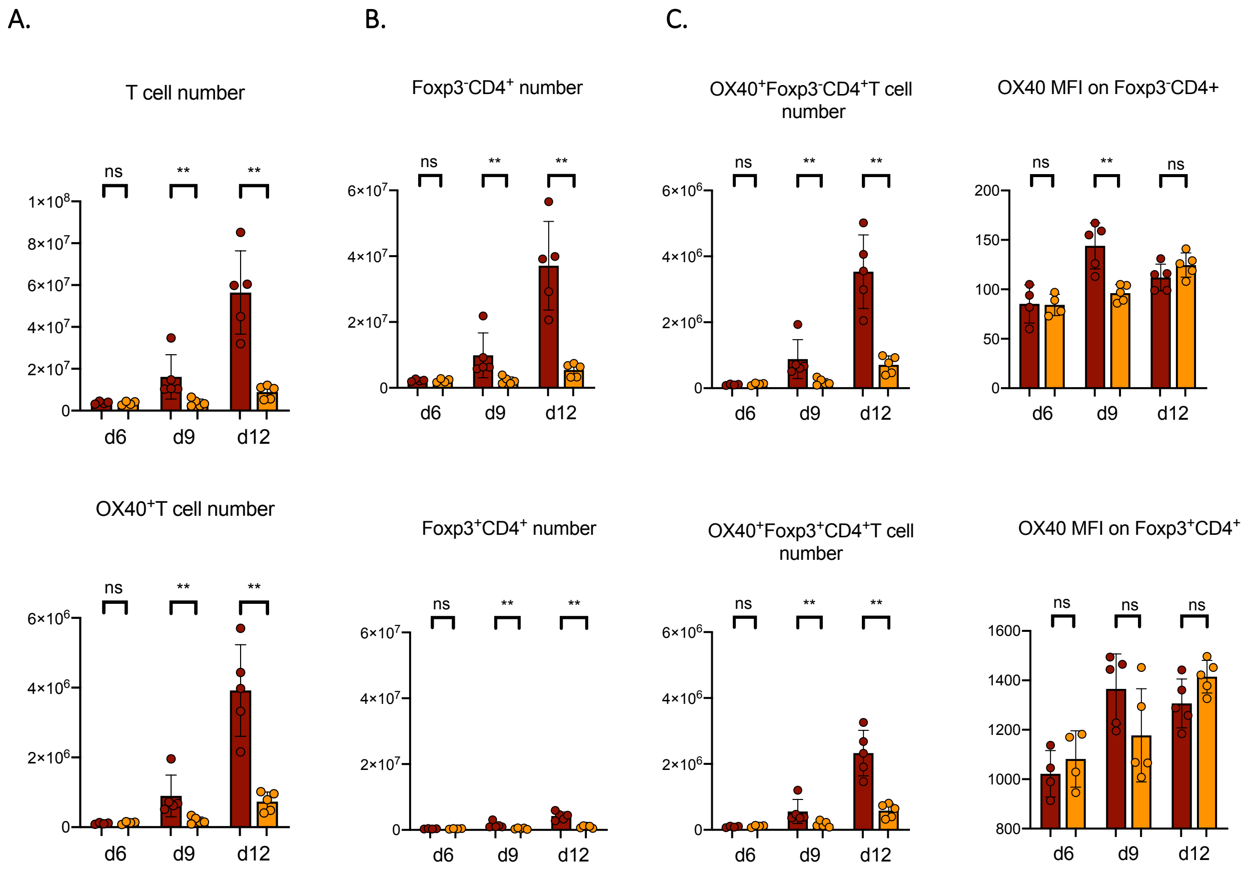

Fig. S3

(A) The number of T cells and OX40$^+$T cells in allo and iso-draining lymph node. **(B)** The number of Foxp3$^-$CD4$^+$ cells and Foxp3$^+$CD4$^+$ cells. **(C)** The number of OX40$^+$cells and expression level of OX40 on each CD4$^+$T cell phenotype.

Video S1

Example video for accepted ear pinna heart graft more than 180 days.
